# Territorial inequities in access to essential medicines for neglected tropical diseases in a decentralised European health system: a nationwide assessment in Spain

**DOI:** 10.1186/s40249-026-01489-8

**Published:** 2026-09-18

**Authors:** Moncef Belhassen-García, Fernando de la Calle-Prieto, José Manuel del Moral Sánchez, Montserrat Alonso-Sardón, Blanca Ayuso-Garcia, Jose-Ramon Blanco, Cristina Bustos Morell, David Campany-Herrero, Cristina Carranza, Miren Ercilla Liceaga, Milagros Garcia López-Hortelano, Elisa García-Vázquez, Beatriz Garrido, Michele Hernandez Cabrera, Carlos Ibero, Aitziber Illaro Uranga, Cristina Jimenez Núñez, Xabier Kortajarena Urkola, Julia Lapeña, Jara Llenas-García, Ana López-Vizcaíno, Irene Losada-Galvan, Paloma Merino, Álvaro Moreno, Ana María Mulet, Miriam Navarro, Javier Pardo Lledías, Jose A. Perez-Molina, Leonor Periañez Parraga, Gracia Picazo Sanchís, Laura Pozo Rosado, Julia Praena-Segovia, Joaquín Salas-Coronas, Fernando Salvador, Joaquín Urda-Romacho, María Romay-Barja

**Affiliations:** 1 Department of Internal Medicine, Infectious Diseases Unit, Salamanca University Hospital, Biomedical Research Institute of Salamanca, Centre for Tropical Diseases Research, University of Salamanca, Salamanca, Spain; 2 Department of Preventive Medicine and Public Health, University of Salamanca, Biomedical Research Institute of Salamanca, Centre for Tropical Diseases Research, University of Salamanca, Salamanca, Spain; 3 Infectious Diseases Unit, Lucus Augusti University Hospital, Lugo, Spain; 4 Department of Infectious Diseases, San Pedro Hospital, Logroño, Spain; 5 Hospital Pharmacy Department, Barbastro Hospital, Barbastro, Spain; 6 Pharmacy Department, Vall d’Hebron University Hospital, Barcelona, Spain; 7 University Institute of Biomedical and Health Research, University of Las Palmas de Gran Canaria, Las Palmas de Gran Canaria, Spain; 8 National Referral Unit for Imported Diseases and International Health, High Level Isolation Unit, IdiPAZ, La Paz-Carlos III University Hospital, CIBERINFEC, Madrid, Spain; 9 Hospital Pharmacy Department, Vega Baja University Hospital, Orihuela, Spain; 10 Pharmacy Department, OSI Debabarrena, Basque Health Service, San Sebastián, Spain; 11 Vega Baja University Hospital, Orihuela, Spain; 12 Department of Clinical Medicine, Miguel Hernández University, Elche, Spain; 13 Infectious Diseases and Tropical Medicine Unit, Insular Maternal and Child University Hospital, University of Las Palmas de Gran Canaria, Las Palmas de Gran Canaria, Spain; 14 Department of Infectious Diseases, Navarra University Hospital, Pamplona, Spain; 15 Pharmacy Department, Marqués de Valdecilla University Hospital, Santander, Spain; 16 Hospital Pharmacy Department, La Paz University Hospital, Madrid, Spain; 17 Department of Infectious Diseases, Donostia University Hospital, Biogipuzkoa Health Research Institute, San Sebastián, Spain; 18 Barcelona Institute for Global Health, Barcelona, Spain; 19 CIBERINFEC, Instituto de Salud Carlos III (ISCIII), Madrid, Spain; 20 Department of Clinical Microbiology, Hospital Clínico San Carlos, Faculty of Medicine, Complutense University of Madrid, Madrid, Spain; 21 Pharmacy Department, Virgen del Puerto Hospital, Extremadura Health Service, Plasencia, Spain; 22 Ministry of Health, Madrid, Spain; 23 Department of Internal Medicine, Marqués de Valdecilla University Hospital, Santander, Spain; 24 National Referral Centre for Tropical Diseases, Department of Infectious Diseases, Biomedical Research Networking Centre for Infectious Diseases, Carlos III Health Institute, Ramón y Cajal University Hospital, Madrid, Spain; 25 Pharmacy Department, Son Espases University Hospital, Mallorca, Spain; 26 Hospital Pharmacy Department, Castile-La Mancha Health Service, Ciudad Real, Spain; 27 Department of Internal Medicine, Virgen del Puerto Hospital, Extremadura Health Service, Plasencia, Spain; 28 Clinical Unit of Infectious Diseases, Microbiology and Parasitology, Instituto de Biomedicina de Sevilla (IBiS), Virgen del Rocío University Hospital, CSIC/University of Seville, Seville, Spain; 29 International Health Research Group of Almería, Faculty of Health Sciences, University of Almería, Almería, Spain; 30 Tropical Medicine Unit, Poniente University Hospital, El Ejido, Almería, Spain; 31 International Health Unit Vall d’Hebron-Drassanes, Infectious Diseases Department, Vall d’Hebron University Hospital, PROSICS Barcelona, Barcelona, Spain; 32 Hospital Pharmacy Department, Poniente University Hospital, Almería, Spain; 33 National Centre for Tropical Medicine, Carlos III Health Institute, Madrid, Spain

**Keywords:** Neglected tropical diseases, Access to medicines, Health equity, Pharmaceutical policy, Decentralized health systems, Europe

## Abstract

**Background:**

Neglected tropical diseases (NTDs) represent an increasing but often overlooked challenge for European health systems owing to international mobility, migration, travel, and climate-related changes in disease ecology. Although effective treatments exist for most NTDs and many are included in the World Health Organization (WHO) Model List of Essential Medicines, their availability in decentralised high-income health systems remains poorly characterised. This study assessed the availability, accessibility, and territorial variability of essential medicines for NTDs across the autonomous communities of Spain and identified the main barriers to equitable access.

**Methods:**

A nationwide, cross-sectional study was conducted across the 17 autonomous communities of Spain. Data were collected between January and December 2025 through a structured survey of Hospital Pharmacy Services in centres with expertise in tropical medicine and infectious diseases and were complemented by expert clinical validation. Medicines were classified according to commercial status, access pathways, dispensing circuits, and regional availability. Descriptive analyses summarised inter-regional variability from a health systems and policy perspective.

**Results:**

Data were obtained for 63 medicines and vaccines, generating 1134 records from all 17 autonomous communities. Twenty-two medicines were commercially available in Spain, whereas 39 required exceptional access mechanisms. Routine stock availability across all centres was reported in only 31.1% of valid records, while 52.8% reported no routine stock and 16.1% restricted availability to reference centres. Joint management by prescribing physicians and hospital pharmacy services was required in 72.1% of access procedures. Marked territorial variability was also observed in dispensing circuits and financing mechanisms, with 47.9% of valid records requiring full out-of-pocket payment and 47.1% involving co-payment.

**Conclusions:**

Access to essential medicines for NTDs in Spain remains structurally fragmented and territorially unequal. Strengthening coordinated national pharmaceutical policies is necessary to reduce reliance on exceptional access mechanisms and ensure equitable, timely, and sustainable access to essential medicines in Spain and other decentralised European health systems.

**Graphical Abstract:**

**Figure:**
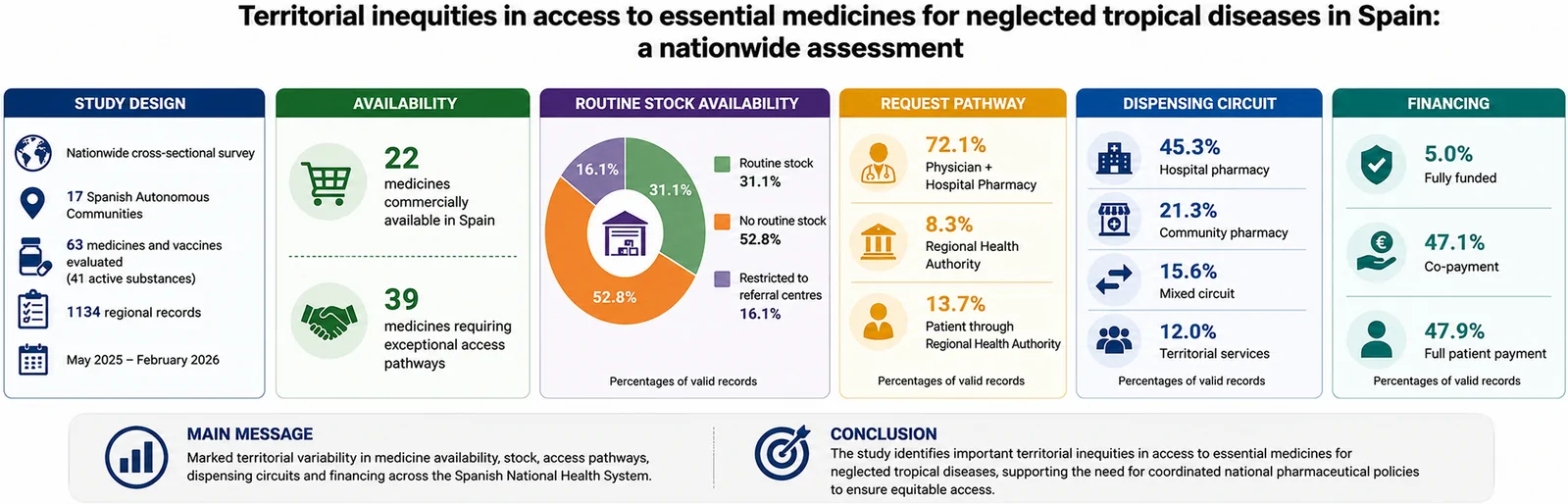

**Supplementary Information:**

The online version contains supplementary material available at https://doi.org/10.1186/s40249-026-01489-8.

## Background

Neglected tropical diseases (NTDs) comprise a heterogeneous group of infectious and parasitic conditions that disproportionately affect socially and economically vulnerable populations and have historically remained peripheral to pharmaceutical markets and health policy priorities. Although their global burden is greatest in low- and middle-income countries, increasing international mobility, migration, travel, and global interconnectedness have rendered NTDs a persistent and growing concern for health systems in high-income European countries [1, 2]. In Spain, most NTDs are regularly diagnosed as imported infections; however, some are autochthonous or zoonotic, underscoring the need for health systems capable of ensuring timely, equitable, and effective access to appropriate treatment [3].

Access to essential medicines is a cornerstone of universal health coverage and a central determinant of health equity [4]. However, access to effective medicines for NTDs remains constrained by limited commercial incentives, fragmented markets and complex regulatory and procurement pathways [5], despite many of these medicines being included in the World Health Organization (WHO) Model List of Essential Medicines. As a result, these medicines are often accessed through exceptional rather than routine mechanisms [6].

In Spain, these challenges occur within a highly decentralised national health system, where national authorities establish the regulatory framework for medicines, whereas procurement, hospital pharmaceutical management, and several access procedures are implemented at regional and local levels [7]. Although this model allows adaptation to local organisational contexts, it may also generate territorial variation in access to medicines. Consequently, medicines that are formally available within the national system may still be subject to heterogeneous access pathways, potentially resulting in avoidable inequities in access to treatment [8].

European Union-level initiatives [9] aimed at strengthening pharmaceutical preparedness, supply security, and cross-country coordination have underscored the importance of identifying national and subnational access gaps that may undermine these objectives [10]. In this context, decentralised health systems, while offering opportunities for context-sensitive and tailored responses, may also give rise to structural inequities if access to essential medicines is not explicitly coordinated and governed. Persistent disparities in access have been described in several decentralised healthcare systems, suggesting that organisational, regulatory and governance factors may influence the availability of low-volume medicines and vaccines [11].

Despite the clinical, ethical, and public health importance of access to NTD treatments, systematic and comparable evidence on real-world availability and access mechanisms in Spain remains limited [3, 4]. Existing information is fragmented and insufficient to inform coordinated national or supranational strategies [12]. To address this gap, we conducted a nationwide assessment of the availability, access mechanisms, and territorial variability of essential medicines and vaccines for NTDs across the Spanish Autonomous Communities using a structured survey of hospital pharmacy services reviewed by experts in tropical medicine and international health.

## Methods

### Study design

A descriptive, cross-sectional, multicenter, nationwide study was conducted between May 2025 and February 2026 to assess the availability and access mechanisms for essential medicines and vaccines used in the treatment of NTDs within the Spanish National Health System (SNHS), from a territorial equity, health systems, and pharmaceutical policy perspective. The analysis was structured by Autonomous Communities (ACs), with the 17 Spanish Autonomous Communities considered the primary unit of analysis. Data from the Autonomous Cities of Ceuta and Melilla were not included.

This study did not evaluate patient-level clinical outcomes, prescribing appropriateness, treatment effectiveness, or medicine utilisation. Its focus was limited to the availability of medicines and vaccines, access mechanisms, procurement pathways, dispensing circuits, and territorial variability across the Spanish National Health System.

### Study setting and participants

The study was clinically led by specialists in NTDs and infectious diseases from the different Spanish ACs, in collaboration with hospital pharmacy services. Within Spain’s decentralised National Health System, organisational models vary across regions. Accordingly, data collection in each AC was coordinated by clinicians with recognised expertise in NTDs, working jointly with hospital pharmacists when appropriate. All contributors had direct operational knowledge of the management, procurement, prescription, and dispensing of NTD-related medicines, ensuring accurate representation of regional access pathways and distribution circuits.

In addition, an expert panel in tropical medicine, comprising members of the Neglected Tropical Diseases Working Group of the Spanish Society of Tropical Medicine and International Health (SEMTSI), reviewed the study variables, assessed the relevance and completeness of the selected medicines and vaccines, and contributed to the contextual interpretation of findings from clinical, public health and health-system perspectives. No patient-level clinical data or clinical outcomes were collected or analysed.

Reference centres were identified through the professional networks of the SEMTSI, national expertise in tropical medicine and infectious diseases, and recognised clinical activity in the diagnosis and management of NTDs. The objective was not to obtain a statistically representative sample of hospitals, but rather to identify centres expected to manage patients requiring access to NTD medicines and vaccines within each AC. Whenever possible, all relevant reference centres known to the study investigators and SEMTSI network were invited to participate.

### Data collection

Data were collected using a structured Excel-based template designed ad hoc by the research team. The instrument was initially piloted in one autonomous community and subsequently standardized for distribution to the remaining ACs.

Supplementary material 1 lists the 63 medicines and the corresponding 41 active ingredients for NTDs available in Spain. For each active substance, immunotherapy, or vaccine relevant to NTD management, the following variables were systematically collected. The definitions of the medicine access pathways used in this study are provided in Table 1**.**

**Table 1:** Variables collected in the standardized data collection template

Domain	Variable
Medicine characteristics	Active substance, immunotherapy, or vaccine
	Main clinical indication
Access pathway	Commercially available in Spain
	Foreign medicine
	Medicine in special situations or other exceptional access mechanisms
Dispensing	Hospital pharmacy
	Community pharmacy
	Mixed dispensing circuit according to indication
Availability	Routine stock availability
	Individual case-by-case request
	Availability restricted to reference centres
Financing and logistics	Qualitative observations on delays, shortages, administrative barriers, or local organisational issues
Contextual information	Autonomous community, completion date, and professional profile of the respondent

Regulatory status, commercial availability, and access pathways were verified and updated through February 2026. Medicines were categorised according to market authorisation, distribution channel (including hospital-only dispensing), and special access mechanisms [such as Medicine in special situations (MSS) or foreign medicine procedures], in order to distinguish formal regulatory status from real-world access conditions within the SNHS (Table 2).

**Table 2:** Definitions of medicine access pathways used in the study

Access pathway	Definition
Commercially available in Spain	Medicinal product authorised and marketed in Spain according to the approved Summary of Product Characteristics (SmPC). Reimbursement depends on the indication and funding conditions of the Spanish National Health System
Foreign medicine	Medicinal product not authorised in Spain but authorised and marketed in another country and imported through the official foreign medicine procedure
Medicine in special situations	Regulatory mechanism allowing access to medicines not routinely marketed in Spain, including named-patient authorisations and other exceptional access procedures established by Spanish pharmaceutical legislation

To enhance comparability across ACs, all participants used the same standardised data collection template, definitions and instructions. Information was reviewed jointly by clinicians and hospital pharmacists whenever possible. When discrepancies were identified, responses were clarified through direct communication with the participating centres and subsequently reviewed by the study coordination team before inclusion in the final dataset.

### Procedures

An Excel-based data collection template was electronically distributed to designated collaborators in each AC. Given the heterogeneity of organisational structures across Spain’s decentralised National Health System, data collection procedures were not uniform and were adapted to the operational model of each AC. In some regions, data were compiled primarily through hospital pharmacy services, whereas in others they were coordinated by clinical teams with expertise in NTDs.

Before nationwide implementation, the template was reviewed by clinicians, hospital pharmacists and members of the SEMTSI Neglected Tropical Diseases Working Group to assess clarity, completeness and practical applicability. Minor modifications were introduced following this review process. Returned questionnaires were checked for completeness and internal consistency. Missing or unclear responses were resolved whenever possible through direct contact with respondents. No formal imputation procedures were performed.

Data were collected in a decentralised manner and subsequently harmonised and reviewed by the coordinating research team. In cases of discrepancies, inconsistencies, or incomplete information, direct clarification was sought from local respondents to ensure internal consistency and comparability. Preliminary results were reviewed during working sessions of the SEMTSI Expert Group with the aims of assessing data consistency, discussing uncertain or discrepant responses, identifying relevant patterns across ACs, and interpreting the findings within the broader context of pharmaceutical governance, tropical medicine practice and public health preparedness.

### Data analysis

A descriptive analysis was performed, focusing on inter-AC comparisons of i) availability of each medicine or vaccine; ii) predominant access mechanisms; and iii) variability in dispensing and access circuits. Quantitative computations were performed using IBM SPSS Statistics for Windows, Version 30.0 (IBM Corp., Armonk, NY, USA) To quantify the differences between regions, absolute and relative measures (counts and percentages) were calculated for each AC. Descriptive statistics (mean, standard deviation, median, interquartile range, and range) were calculated to explore the variability in price per container or unit among the ACs. Results were presented using comparative tables and territorial availability maps. Given the exploratory, policy-oriented, and descriptive nature of the study, no inferential analyses were performed.

## Results

This study compiled basic data on the 63 medicines and vaccines related to NTDs available in Spain, covering 41 active ingredients essential for the treatment of these diseases (see Table 3).

**Table 3:** Basic characteristics of 63 medicines and vaccines related to neglected tropical diseases

NTD medicines and vaccines	*n* = 63
Active substances/Immunotherapy/Vaccine	41
Route of administration	
Oral	40
Intravenous	16
Intramuscular	3
Subcutaneous	1
Topical	1
Intravenous/Intramuscular/Intralesional	1
Type of access	
Special Situations Medicine (foreign)	39
Marketed in Spain	22
No data	2
Special Situations Medicine processing	
Special authorisation	28
Special authorisation with manufacturer agreement	1
Stock availability	10
Package price (€)	
Mean (± SD)	304.90 (± 769.96)
Median (P25–P75)	33.56 (6.48–159.40)
Range (min–max)	1.66–3952.00

Twenty-two of these are marketed in Spain, while 39 correspond to medicines for special situations/abroad. All 17 ACs (two centers in the AC of Madrid) participated by reporting on the 63 NTD-related medicines, obtaining a total of 1134 records. Overall quantitative descriptive data on medicine availability, access pathways, processing requirements, dispensing circuits and patient costs are summarised in Table 4.

**Table 4:** Availability, access pathways, financing mechanisms, processing requirements and dispensing circuits of essential medicines and vaccines for neglected tropical diseases across 1134 records from the Spanish Autonomous Communities

	*n*	%	% valid
Stock availability			
Yes, in all centres	310	27.3	31.1
Yes, in reference centres	161	14.2	16.1
No	526	46.4	52.8
ND	137	12.1	—
Cost to the user (administration contribution to payment)			
Free of charge (full contribution)	33	2.9	5.0
Co-payment (reduced contribution)	312	27.5	47.1
Full payment (no contribution)	317	28.0	47.9
ND	472	41.6	—
Request processing			
Physician with hospital pharmacy	405	35.7	72.1
Physician with regional health authority	47	4.1	8.3
Patient with hospital pharmacy	33	2.9	5.9
Patient with regional health authority	77	6.8	13.7
ND	562	52.5	—
Dispensing site			
Hospital pharmacy	466	41.2	45.3
Community pharmacy	219	19.3	21.3
Community or hospital pharmacy, depending on circumstances	160	14.1	15.6
Territorial service	123	10.8	12.0
Other	60	5.3	5.8
ND	106	9.3	—

Overall quantitative descriptive data demonstrated substantial heterogeneity in medicine availability, access pathways, financing mechanisms, processing requirements, dispensing circuits, and patient costs across the Spanish Autonomous Communities (Table 4). More than half of the evaluated records indicated the absence of routine stock availability, whereas access restricted to designated reference centres was also common. Exceptional procurement mechanisms, including foreign medicine procedures and other special access pathways, represented a major component of access for many essential medicines and vaccines for NTD.

### Overall availability of medicines and vaccines for NTDs

The analysis revealed marked territorial heterogeneity in the availability of medicines and vaccines for the treatment of NTDs. None of the ACs evaluated ensured systematic and homogeneous access to the full set of medicines considered essential.

The availability of treatments did not follow a uniform pattern and was not clearly associated with population size, healthcare activity, or the presence of tertiary referral centres, but rather depended primarily on organisational factors, local pharmaceutical management circuits, and prior institutional experience.

### Types of access and procurement mechanisms

A substantial proportion of the medicines analysed were not commercially available in Spain and required exceptional access mechanisms, predominantly through foreign medicines or special-use procedures (Table 3). This pattern was consistently observed across most ACs and affected antiparasitic treatments, vaccines, and antivenoms alike. For example, benznidazole and nifurtimox were frequently accessed through foreign medicine procedures, while certain snake antivenoms and dengue vaccine required ad hoc procurement requests in several regions. In line with this, stock availability data showed that only 31.1% of valid records reported routine availability across all centres within the AC, whereas 52.8% reported no routine stock and 16.1% indicated availability restricted to reference centres. Reliance on these exceptional pathways showed considerable inter-regional variability, with relevant differences in administrative procedures, the existence of prior stock, and estimated time to acquisition. In several ACs, access to certain essential medicines was conditional on individual, case-by-case requests, without guarantees of immediate availability; in some cases, medicines were available only in one designated reference centre within the AC. This administrative complexity is reflected in request-processing data, where 72.1% of valid records required joint management between the prescribing physician and hospital pharmacy services, 8.3% involved regional health authorities, and 13.7% required direct patient interaction with regional authorities.

**Fig. 1:**
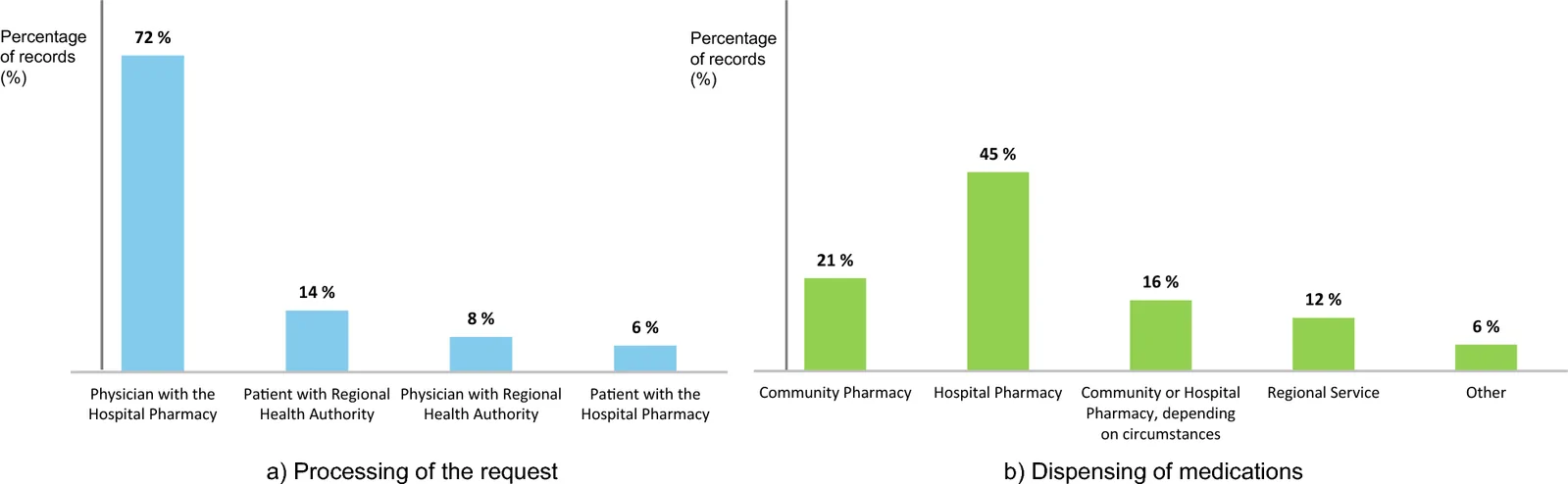
Distribution of request processing mechanisms and medicine dispensing circuits for essential medicines and vaccines for neglected tropical diseases across the Spanish Autonomous Communities. **a** Request processing pathways. **b** Medicine dispensing circuits

The management of medicine requests and pharmaceutical service provision was predominantly coordinated through hospital pharmacy services, although substantial variability was observed in request processing and dispensing circuits across the Spanish Autonomous Communities (Fig. 1). Detailed distributions of request processing and medicine dispensing are presented in panels a and b, respectively.

### Centre-level distribution and intra-regional inequities

**Fig. 2:**
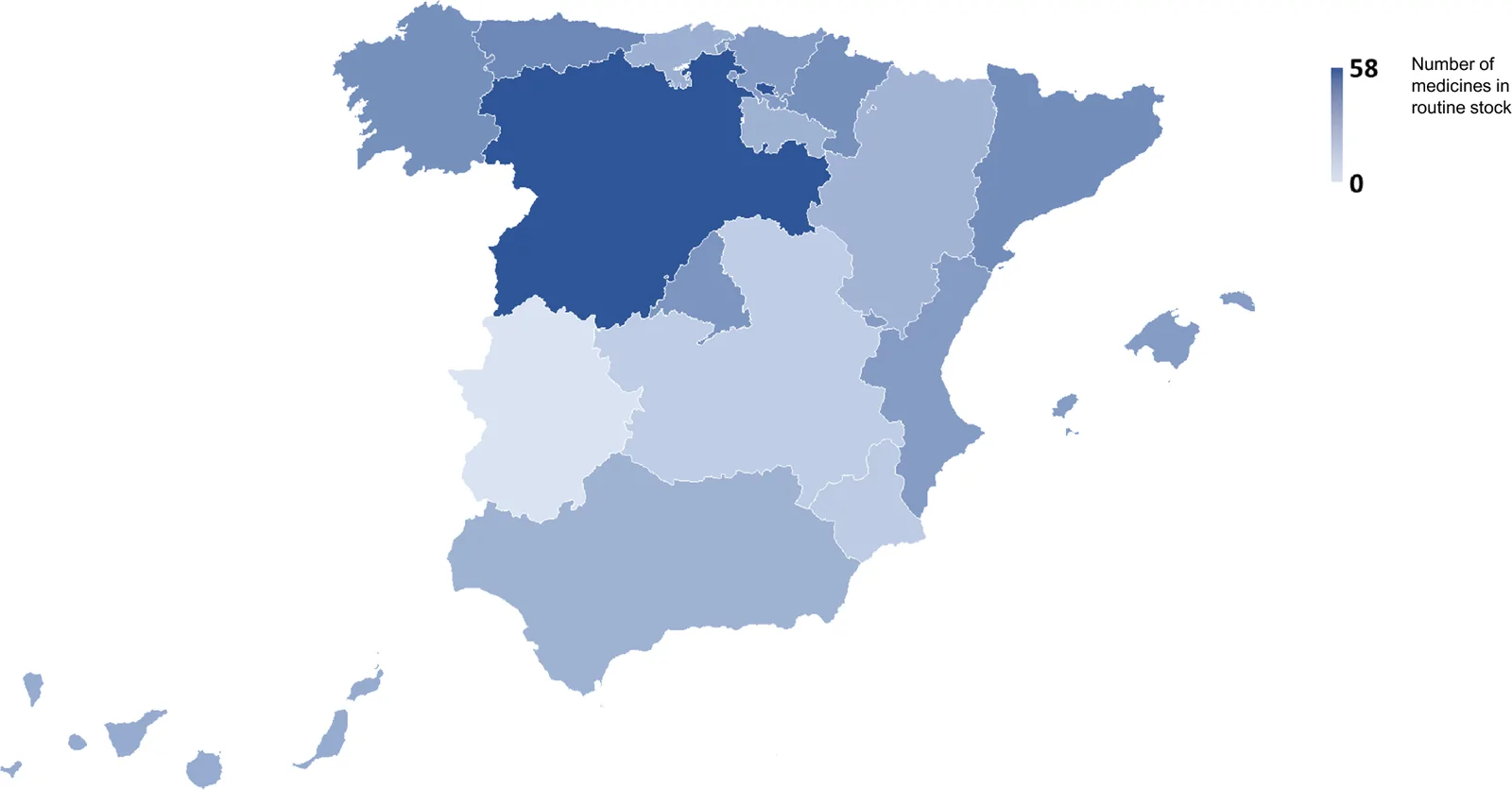
Number of neglected tropical diseases-related medicines in stock (in all centers/in referral centers) by Autonomous Communities

The map of Spain in Fig. 2 illustrates the degree of availability of essential medicines for NTDs across the ACs. Castilla y León showed the highest number of essential medicines available in routine stock, whereas several other ACs reported substantially lower levels of availability, particularly for non-commercialised antiparasitic agents, certain vaccines, and antivenoms, consistent with the finding that more than half of valid stock records (52.8%) indicated absence of routine availability. Detailed data by AC are presented in Supplementary Table S2.

Recurrently, the availability of medicines for NTDs was concentrated in a limited number of designated reference centres, even within the same AC. For example, in some regions, specific treatments such as antivenoms or foreign-imported antiparasitic drugs were available in only one tertiary hospital, requiring internal referrals from other hospitals within the same AC. This concentration is consistent with the 16.1% of valid records reporting availability restricted to reference centres.

This pattern was observed both in highly populated ACs (including regions with multiple tertiary hospitals) and in smaller ACs, where stock concentration in a single centre was more pronounced. In several cases, hospitals within the same health area reported different access circuits for the same medicine, reflecting organisational rather than epidemiological variability. In many settings, actual access to treatment depended on local knowledge of administrative procedures and established professional networks.

### Cost to the user/financing

**Fig. 3:**
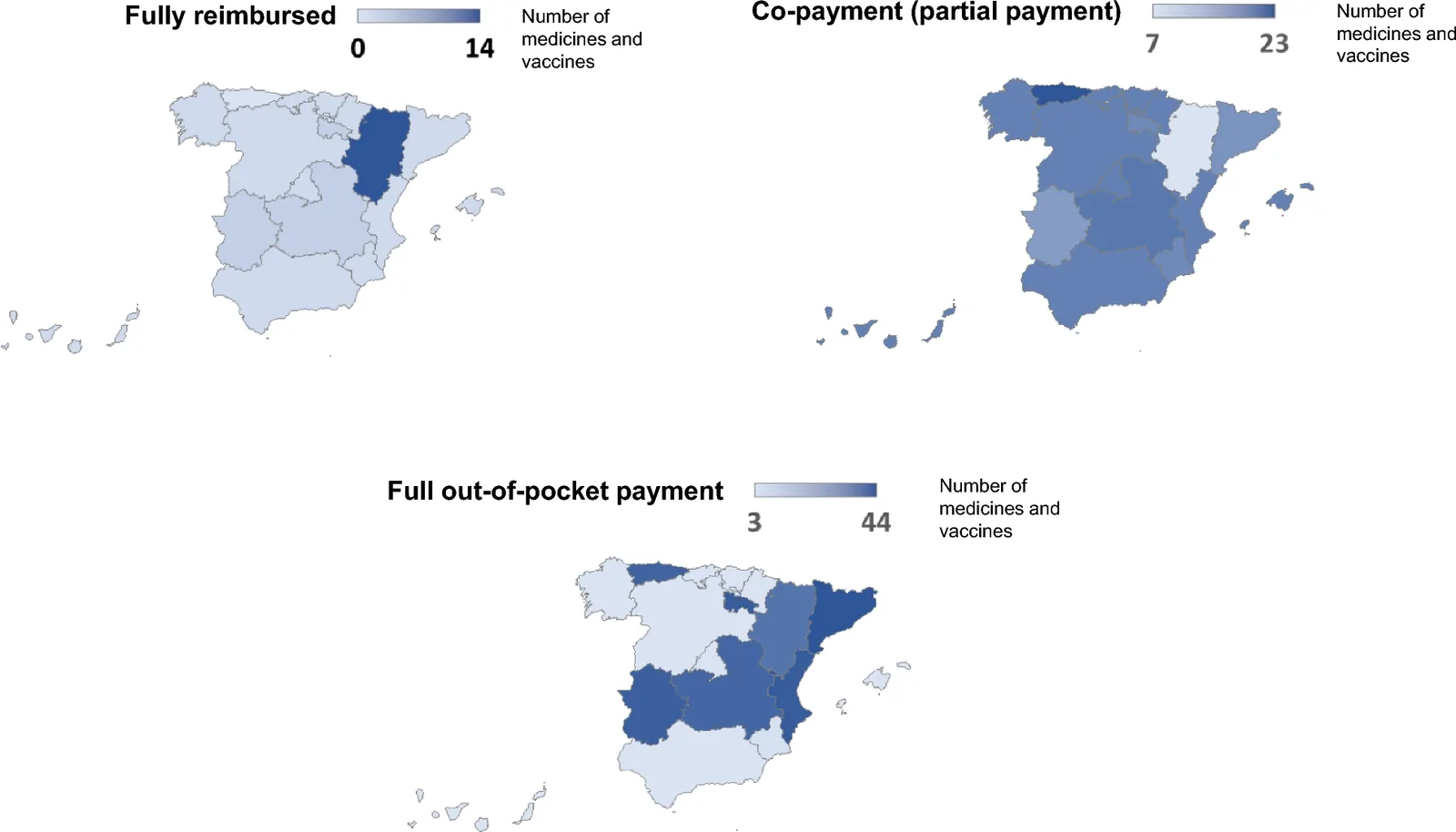
Cost to the user: distribution maps (number of medicines per autonomous communities)

Regional variability in patient financing mechanisms for essential medicines and vaccines for neglected tropical diseases is illustrated across the 17 Spanish ACs using three choropleth maps, each displaying the frequency of a specific patient payment category. The corresponding geographic distributions are shown in Fig. 3. Detailed financing data for each AC are presented in Supplementary Table S3.

Marked inter-regional differences were observed. In several ACs, NTD-related medicines were fully financed and dispensed through hospital pharmacy services at no direct cost to patients, whereas in others certain medicines obtained through foreign medicine procedures involved partial or full out-of-pocket payment or individual reimbursement processes. Among valid financing records, only 5.0% of medicines were reported as fully financed, while 47.1% involved co-payment and 47.9% required full out-of-pocket payment. Differences were particularly evident for antiparasitic treatments accessed through exceptional pathways and for selected vaccines not routinely integrated into hospital formularies.

These findings indicate the absence of a uniform national financing model, with variability in whether costs were absorbed at hospital level or transferred, at least temporarily, to patients.

### Vaccines and antivenoms with high clinical impact

Vaccines and antivenoms included in the analysis -such as rabies vaccine, dengue vaccine, and snake antivenoms- showed particularly relevant limitations in availability. In numerous ACs, these products were not routinely stocked, and access depended on exceptional procurement, often involving explicit logistical and economic barriers, including high acquisition costs or minimum order requirements.

### Identified structural barriers

A descriptive summary of barriers reported by survey participants identified recurrent non-clinical constraints affecting access to NTD treatments within the Spanish National Health System. Frequently mentioned challenges included delays in medicine acquisition, uncertainty regarding administrative pathways, reliance on ad hoc import procedures, and the absence of stable and predictable supply strategies. These issues were reported by professionals across multiple ACs, indicating that they are not isolated events but rather reflect broader structural access constraints.

### Synthesis of findings

Overall, the results indicate that the availability of medicines and vaccines for NTDs in Spain is heterogeneous, fragmented, and highly dependent on exceptional access mechanisms, with marked differences between autonomous communities and between centres within the same community. This variability is further illustrated by dispensing circuits, with 45.3% of valid records indicating hospital pharmacy-only dispensing, 21.3% community pharmacy dispensing, 15.6% mixed circuits, and 12.0% territorial service distribution. This variability introduces avoidable territorial inequities and limits the capacity of the SNHS to guarantee equitable and timely access to essential treatments for NTDs.

## Discussion

This nationwide assessment demonstrates that access to essential medicines and vaccines for neglected tropical diseases within the Spanish National Health System remains territorially heterogeneous and structurally fragmented. Three principal findings emerged. First, availability varied substantially across the 17 Spanish ACs, with no region ensuring systematic access to the full range of essential treatments. Second, many medicines relied on exceptional procurement mechanisms rather than routine pharmaceutical pathways[6]. Third, access was frequently concentrated in designated reference centres, creating both inter- and intra-regional variability. Together, these findings indicate that equitable and timely access to essential NTD medicines continues to depend largely on regional organisational practices rather than on a coordinated national pharmaceutical strategy [4].

The decentralised organisation of the Spanish health system, while enabling responsiveness to local epidemiological and organisational needs, appears to amplify vulnerabilities inherent to medicines characterised by low commercial attractiveness and high regulatory complexity [5, 12]. In the absence of a coordinated national strategy for NTD medicines, responsibility for ensuring access is devolved to local administrative circuits, reference centres, and, at times, the individual initiative of healthcare professionals. This model generates avoidable territorial inequities and places patients in situations of vulnerability determined more by place of care than by clinical need, in line with prior analyses of inequity in decentralised health systems [8]. The fragmentation observed even within large ACs reinforces the interpretation of this problem as systemic rather than regional [12]

Similar challenges have been reported in other high-income countries, where limited commercial incentives and reliance on exceptional procurement mechanisms have compromised timely access to essential antiparasitic medicines [5, 10].

These findings suggest that improving equitable access to essential NTD medicines will require more coordinated governance and procurement strategies rather than continued reliance on exceptional access pathways.A particularly important finding was the limited availability of vaccines and antivenoms with high clinical and public health value, including rabies vaccine, dengue vaccine, and snake antivenoms. In many ACs, these products were not routinely stocked and depended on exceptional procurement mechanisms, potentially delaying access in time-sensitive clinical situations. This finding extends beyond neglected tropical diseases and highlights broader challenges in pharmaceutical preparedness and health system resilience, particularly in the context of increasing international travel, migration, and climate-related changes in disease distribution [13–16]. Ensuring timely access to these products should therefore be considered a key component of national preparedness strategies. From a health policy perspective, these results suggest that access to NTD medicines in Spain is shaped more by administrative and market logics than by principles of universality, equity, clinical need, or public health priority, reflecting tensions previously described in analyses of access to essential medicines [4, 10]. The normalisation of exceptional access pathways and the marked regional variability in financing mechanisms reflect a disconnect between the formal recognition of these medicines as essential and their practical integration into the benefit package of the Spanish National Health System. Consequently, patients may face different financial barriers according to their AC rather than their clinical needs. Addressing this gap requires structural pharmaceutical policies that ensure stable, predictable, and equitable access to essential NTD medicines [9].

Potential strategies include the establishment of centralised procurement and negotiation mechanisms, the development of national or supranational strategic stockpiles, the formal designation of reference centres with transparent access circuits, and the explicit integration of NTD medicines into national pharmaceutical policy frameworks [12].

An important interpretative consideration is the inherently dynamic nature of both medicine availability and hospital-level pharmaceutical management within decentralised health systems. Access to essential but low-volume medicines is not static; it may change substantially over relatively short periods in response to regulatory decisions, procurement strategies, supply agreements, and adaptations in hospital pharmacy circuits. For this reason, any assessment requires the definition of a temporal cut-off. In the present study, data collection was anchored to the year 2025, providing a coherent snapshot of access conditions at a specific point in time.

Medicine availability is dynamic and may change rapidly following regulatory, commercial, or procurement decisions. Although access to some medicines improved during the study period, these advances remain dependent on pharmaceutical governance and may not ensure sustained equitable access across ACs. This reinforces the need for continuous monitoring and coordinated national procurement strategies.

Improving access to essential medicines for NTDs must be framed within the broader challenge of guaranteeing equitable healthcare for migrants, asylum seekers, refugees, and other vulnerable populations, who are disproportionately affected by these diseases [17]. Healthcare entitlement alone does not guarantee effective access to treatment. Ensuring equitable access to essential NTD medicines will require coordinated national strategies, rapid access mechanisms, and sustained pharmaceutical preparedness across regions.

This study has limitations. Information was based on surveys completed by Hospital Pharmacy Services and expert interpretation, which may not capture all temporal fluctuations in medicine availability. In addition, the cross-sectional and descriptive design allows identification of patterns and inter-regional differences but does not permit causal inference or direct estimation of the economic and clinical impact of the identified barriers, which should be addressed in future research. Despite these limitations, the consistency of findings across ACs supports the robustness of the conclusions. Beyond simple description, this study characterises administrative, logistical, and organisational barriers, providing policy-relevant evidence to inform strategies aimed at improving equitable access [10].

Although this study focuses on Spain, the challenges identified are relevant to other decentralised European healthcare systems facing similar tensions between regional autonomy, pharmaceutical governance and equitable access to low-volume medicines. From a Universal Health Coverage perspective, ensuring access to essential NTD medicines represents not only a clinical necessity but also a measure of health-system equity and preparedness. Current European initiatives aimed at strengthening pharmaceutical resilience and reducing medicine shortages provide a favourable framework for developing coordinated approaches to these neglected but essential medicines.

In conclusion, this nationwide study demonstrates that ensuring equitable access to essential medicines for neglected tropical diseases remains a significant challenge in decentralised healthcare systems, even within a high-income country with universal health coverage. The findings indicate that access is determined not only by medicine availability but also by the organisation of pharmaceutical governance, procurement processes, and administrative pathways, all of which can generate avoidable territorial inequities.

Beyond the Spanish context, this study provides evidence that access to low-demand but clinically essential medicines should be regarded as a core component of health-system preparedness and pharmaceutical resilience. Strengthening coordinated procurement, harmonising access procedures, and integrating these medicines into routine pharmaceutical policies are likely to improve equity, reduce delays in treatment, and enhance preparedness for imported and emerging diseases. These findings may inform future pharmaceutical policies and governance strategies in Spain and in other decentralised healthcare systems facing similar organisational challenges.

## Supplementary Information

Additional file 1

Additional file 2

Additional file 3

## Data Availability

The datasets generated and/or analysed during the current study are available from the corresponding author on reasonable request. Aggregated data supporting the findings are included within the article and its supplementary materials.

## Abbreviations

AC: Autonomous community
MSS: Medicines in special situations
NTD: Neglected tropical disease
SEMTSI: Spanish society of tropical medicine and international health
SmPC: Summary of product characteristics
SNHS: Spanish national health system
WHO: World Health Organization
