# Supplementary material for: Territorial inequities in access to essential medicines for neglected tropical diseases in a decentralised European health system: a nationwide assessment in Spain
Source: Infect Dis Poverty. 2026 Sep 18;15:104. doi: 10.1186/s40249-026-01489-8 (PMC13587272; doi:10.1186/s40249-026-01489-8)
Supplement: Supplementary file 1 [file 40249_2026_1489_MOESM1_ESM.docx]

**Supplementary Table 1. Medicines, immunotherapies and vaccines for neglected tropical diseases (NTDs) and their regulatory status and access pathways within the Spanish National Health System (SNHS).**

| Active Ingredient / Immunotherapy / Vaccine | Pharmaceutical Product / Brand Name (Pack Size) | Route | Regulatory Status in Spain | Access Pathway within SNHS | Processing Modality (if MSS) | RRP (€ VAT included) | Indication(s) |
| --- | --- | --- | --- | --- | --- | --- | --- |
| Albendazole | ESKAZOLE 400 mg tablets (60) | Oral | Marketed in Spain | Standard distribution | Not applicable | 92.76 | Soil-transmitted helminthiasis; LF; Echinococcosis; Trichinosis; Taeniasis/Cysticercosis |
| Amphotericin B | FUNGIZONE 50 mg vial (1) | IV | Not marketed in Spain | MSS – Foreign medicine | Case-by-case authorisation | Not available | Leishmaniasis; Deep mycoses |
| Amphotericin B | AMPHO-MORONAL 100 mg/mL oral suspension (30 mL) | Oral | Not marketed in Spain | MSS – Foreign medicine | Case-by-case authorisation | Not available | Deep mycoses |
| Liposomal Amphotericin B | AMBISOME 50 mg vial (10) | IV | Marketed in Spain | Hospital dispensing only | Not applicable | 1410.78 | Leishmaniasis |
| Meglumine antimoniate | GLUCANTIME 1500 mg/5 mL ampoule (10) | IV/IM/Intralesional | Marketed in Spain | Hospital dispensing only | Not applicable | 15.99 | Leishmaniasis |
| Artemether-lumefantrine | RIAMET 20/120 mg tablets (24) | Oral | Not marketed in Spain | MSS – Foreign medicine | Case-by-case authorisation | Not available | Malaria |
| Artesunate | DUNATE 60 mg vial (1) | IV | Not marketed in Spain | MSS – Foreign medicine | Stock | Not available | Malaria |
| Artesunate | FALCI CARE 60 mg vial (1) | IV | Not marketed in Spain | MSS – Foreign medicine | Stock | Not available | Malaria |
| Azithromycin | AZITHROMYCIN 500 mg tablets (3) | Oral | Marketed in Spain | Standard distribution | Not applicable | 5.62 | Trachoma; Yaws |
| Azithromycin | AZITHROMYCIN 500 mg oral sachet (4) | Oral | Marketed in Spain | Standard distribution | Not applicable | 5.62 | Trachoma; Yaws |
| Azithromycin | AZITHROMYCIN 200 mg/5 mL suspension (30 mL) | Oral | Marketed in Spain | Standard distribution | Not applicable | 6.03 | Trachoma; Yaws |
| Azithromycin | ZITROMAX 500 mg vial (1) | IV | Marketed in Spain | Hospital dispensing only | Not applicable | 22.76 | Trachoma; Yaws |
| Benznidazole | ABARAX 50 mg tablets (100) | Oral | Not marketed in Spain | MSS – Foreign medicine | Case-by-case authorisation | Not available | Chagas disease |
| Benznidazole | ABARAX 100 mg tablets (100) | Oral | Not marketed in Spain | MSS – Foreign medicine | Case-by-case authorisation | Not available | Chagas disease |
| Clofazimine | LAMPRENE 100 mg capsules (100) | Oral | Not marketed in Spain | MSS – Foreign medicine | Case-by-case authorisation | Not available | Leprosy |
| Dapsone + Rifampicin + Clofazimine | MDT-COMBI capsules (360) | Oral | Not marketed in Spain | MSS – Foreign medicine | Case-by-case authorisation | Not available | Leprosy |
| Dapsone | DAPSONE FATOL 50 mg tablets (50) | Oral | Not marketed in Spain | MSS – Foreign medicine | Stock | Not available | Leprosy |
| Dapsone | DAPSON TILLOMED 50 mg tablets (28) | Oral | Not marketed in Spain | MSS – Foreign medicine | Case-by-case authorisation | Not available | Leprosy |
| Diethylcarbamazine (DEC) | SUPATONIN 50 mg tablets (100) | Oral | Not marketed in Spain | MSS – Foreign medicine | Case-by-case authorisation | Not available | Lymphatic filariasis |
| Doxycycline | DOXYCYCLINE 100 mg capsules (14) | Oral | Marketed in Spain | Standard distribution | Not applicable | 2.50 | Lymphatic filariasis |
| Doxycycline | VIBRACINA 50 mg/5 mL suspension (60 mL) | Oral | Marketed in Spain | Standard distribution | Not applicable | 2.50 | Lymphatic filariasis |
| Doxycycline | VIBRAVENOSA 100 mg ampoule (1) | IV | Marketed in Spain | Hospital dispensing only | Not applicable | 2.54 | Lymphatic filariasis |
| Eflornithine | ORNIDYL 200 mg/mL vial (50 mL) | IV | Not marketed in Spain | MSS – Foreign medicine | Case-by-case authorisation | Not available | Human African trypanosomiasis |
| Fexinidazole | No commercial presentation identified | Oral | Not marketed in Spain | Not available during study period | Not applicable | Not available | Human African trypanosomiasis |
| Itraconazole | ITRACONAZOLE 100 mg capsules (14) | Oral | Marketed in Spain | Standard distribution | Not applicable | 12.99 | Mycetoma; Deep mycoses |
| Itraconazole | ODAN 10 mg/mL solution (150 mL) | Oral | Not marketed in Spain | MSS – Foreign medicine | Case-by-case authorisation | Not available | Mycetoma |
| Itraconazole | FUNGOSPOR 10 mg/mL ampoule (25 mL) | IV | Not marketed in Spain | MSS – Foreign medicine | Stock | Not available | Deep mycoses |
| Ivermectin | IVERMECTIN 3 mg tablets (4) | Oral | Marketed in Spain | Standard distribution | Not applicable | 17.48 | Onchocerciasis; LF; Scabies; Strongyloidiasis |
| Ketoconazole | KETOCONAZOLE HRA 200 mg tablets (60) | Oral | Not marketed in Spain | MSS – Foreign medicine | Case-by-case authorisation | Not available | Mycetoma |
| Melarsoprol | ARSOBAL 180 mg/5 mL ampoules | IV | Not marketed in Spain | MSS – Foreign medicine | Case-by-case authorisation | Not available | Human African trypanosomiasis |
| Mebendazole | LOMPER 100 mg tablets (6) | Oral | Marketed in Spain | Standard distribution | Not applicable | 4.40 | Soil-transmitted helminths; Trichinosis |
| Mebendazole | LOMPER 20 mg/mL suspension (30 mL) | Oral | Marketed in Spain | Standard distribution | Not applicable | 4.40 | Soil-transmitted helminths |
| Mefloquine | LARIAM 250 mg tablets (8) | Oral | Not marketed in Spain | MSS – Foreign medicine | Stock | Not available | Malaria |
| Mepacrine | MEPACRINE HYDROCHLORIDE 100 mg tablets (50) | Oral | Not marketed in Spain | MSS – Foreign medicine | Case-by-case authorisation | Not available | Giardiasis |
| Miltefosine | IMPAVIDO 50 mg capsules (56/57) | Oral | Not marketed in Spain | MSS – Foreign medicine | Case-by-case authorisation | Not available | Leishmaniasis |
| Nifurtimox | LAMPIT 120 mg capsules (100) | Oral | Not marketed in Spain | MSS – Foreign medicine | Centralised stock (improved during study period) | Not available | Chagas disease |
| Nitazoxanide | PARAMIX 500 mg tablets (6) | Oral | Not marketed in Spain | MSS – Foreign medicine | Case-by-case authorisation | Not available | Intestinal helminths and protozoa |
| Nitazoxanide | PARAMIX suspension (60 mL) | Oral | Not marketed in Spain | MSS – Foreign medicine | Case-by-case authorisation | Not available | Intestinal helminths and protozoa |
| Paromomycin | PAROMOMYCIN ampoules (2 mL) | IV | Not marketed in Spain | MSS – Foreign medicine | Case-by-case authorisation | Not available | Leishmaniasis |
| Paromomycin | HUMATIN 250 mg capsules (8) | Oral | Marketed in Spain | Standard distribution | Not applicable | 6.24 | Amebiasis |
| Pentamidine | PENTACARINAT 300 mg vial | IV | Marketed in Spain | Hospital dispensing only | Not applicable | 16.00 | Human African trypanosomiasis |
| Permethrin | PERMETHRIN 50 mg/g cream (40 g) | Topical | Marketed in Spain | Standard distribution | Not applicable | 9.73 | Scabies |
| Piperaquine-artenimol | EURARTESIM tablets | Oral | Marketed in Spain | Standard distribution | Not applicable | 59.99 | Malaria |
| Praziquantel | BILTRICIDE / CISTICID 600 mg tablets | Oral | Not marketed in Spain | MSS – Foreign medicine | Stock availability (variable by region) | Not available | Schistosomiasis; Taeniasis/Cysticercosis |
| Primaquine | PRIMAQUINE PHOSPHATE tablets | Oral | Not marketed in Spain | MSS – Foreign medicine | Case-by-case authorisation | Not available | Malaria |
| Quinine | QUININE SULPHATE 300 mg tablets | Oral | Not marketed in Spain | MSS – Foreign medicine | Case-by-case authorisation | Not available | Malaria |
| Rifampicin | RIFAMPICIN capsules/suspension/vial | Oral/IV | Marketed in Spain | Standard distribution | Not applicable | 8.37 | Leprosy; Buruli ulcer |
| Suramin | GERMANIN 1 g vial | IV | Not marketed in Spain | MSS – Foreign medicine | Case-by-case authorisation | Not available | Human African trypanosomiasis |
| Tetracycline | AMBRAMICINA 250 mg capsules (16) | Oral | Not marketed in Spain | MSS – Foreign medicine | Stock | Not available | Trachoma |
| Triclabendazole | EGATEN 250 mg tablets (4) | Oral | Not marketed in Spain | MSS – Foreign medicine | Case-by-case authorisation | Not available | Fascioliasis |
| Rabies immunotherapy | BERIRAB / HYPERRAB | IM | Not marketed in Spain | MSS – Foreign medicine | Stock | Not available | Rabies immunoglobulin |
| Rabies vaccine | RABIPUR 2.5 IU vial | IM | Marketed in Spain | Hospital dispensing only | Not applicable | 77.63 | Rabies |
| Rabies vaccine | VERORAB 2.5 IU vial | IM | Not marketed in Spain | MSS – Foreign medicine | Stock | Not available | Rabies |
| Dengue vaccine | QDENGA 0.5 mL vial | SC | Not marketed in Spain (during study period) | MSS – Special access | Stock (regional variability) | Not available | Dengue |
| Vipera aspis/Berus antivenom | VIPERFAV ampoule | IV | Not marketed in Spain | MSS – Foreign medicine | Stock | Not available | Snake bite |
| Viperidae/Elapidae antivenom | INOSERP PANAFRICA vial | IV | Not marketed in Spain | MSS – Foreign medicine | Case-by-case authorisation | Not available | Snake bite |

*Methodological note: Access status reflects the regulatory and distribution situation within the SNHS during May 2025–February 2026. Medicines were classified according to market authorisation, access pathway, and processing modality.*
