# Supplementary material for: Territorial inequities in access to essential medicines for neglected tropical diseases in a decentralised European health system: a nationwide assessment in Spain
Source: Infect Dis Poverty. 2026 Sep 18;15:104. doi: 10.1186/s40249-026-01489-8 (PMC13587272; doi:10.1186/s40249-026-01489-8)
Supplement: Supplementary file 2 [file 40249_2026_1489_MOESM2_ESM.docx]

**Supplementary Table S2.** Number of centers with neglected tropical disease (NTD)-related medicines in stock by Autonomous Community.

| Autonomous Community | Centers with NTD-related medicines in stock, n |
| --- | --- |
| Andalucía | 20 |
| Aragón | 19 |
| Asturias | 37 |
| Baleares | 29 |
| Canarias | 23 |
| Cantabria | 21 |
| Castilla-La Mancha | 9 |
| Castilla y León | 58 |
| Cataluña | 35 |
| Extremadura | 0 |
| Galicia | 34 |
| Madrid | 32 |
| Murcia | 10 |
| Navarra | 34 |
| País Vasco | 29 |
| La Rioja | 20 |
| Comunidad Valenciana | 29 |
