# Supplementary material for: Territorial inequities in access to essential medicines for neglected tropical diseases in a decentralised European health system: a nationwide assessment in Spain
Source: Infect Dis Poverty. 2026 Sep 18;15:104. doi: 10.1186/s40249-026-01489-8 (PMC13587272; doi:10.1186/s40249-026-01489-8)
Supplement: Supplementary file 3 [file 40249_2026_1489_MOESM3_ESM.docx]

**Supplementary Table S3.** User cost for neglected tropical disease (NTD)-related medicines by Autonomous Community.

| Autonomous Community | Free (full contribution), n | Copayment (partial payment), n | Full payment (no contribution), n |
| --- | --- | --- | --- |
| Andalucía | 1 | 18 | 3 |
| Aragón | 14 | 7 | 35 |
| Asturias | 0 | 23 | 40 |
| Baleares | 1 | 18 | 3 |
| Canarias | 1 | 18 | 3 |
| Cantabria | 1 | 18 | 3 |
| Castilla-La Mancha | 2 | 19 | 39 |
| Castilla y León | 1 | 18 | 3 |
| Cataluña | 1 | 16 | 44 |
| Extremadura | 2 | 15 | 41 |
| Galicia | 1 | 18 | 3 |
| Madrid | 1 | 18 | 3 |
| Murcia | 1 | 17 | 4 |
| Navarra | 1 | 18 | 3 |
| País Vasco | 1 | 18 | 3 |
| La Rioja | 2 | 17 | 42 |
| Comunidad Valenciana | 1 | 18 | 42 |
